# Oxidative damage mediates the association between polycyclic aromatic hydrocarbon exposure and lung function

**DOI:** 10.1186/s12940-020-00621-x

**Published:** 2020-07-02

**Authors:** Limin Cao, Yun Zhou, Aijun Tan, Tingming Shi, Chunmei Zhu, Lili Xiao, Zhuang Zhang, Shijie Yang, Ge Mu, Xing Wang, Dongming Wang, Jixuan Ma, Weihong Chen

**Affiliations:** 1 Department of Occupational & Environmental Health, School of Public Health, Tongji Medical College, Huazhong University of Science and Technology, Wuhan, 430030 Hubei China; 2 Key Laboratory of Environment and Health, Ministry of Education & Ministry of Environmental Protection, and State Key Laboratory of Environmental Health (Incubating), School of Public Health, Tongji Medical College, Huazhong University of Science and Technology, Wuhan, 430030 Hubei China; 3 Zhuhai Center for Disease Control and Prevention, Zhuhai, 519000 Guangdong China; 4 Hubei Center for Disease Control and Prevention, Wuhan, 430079 Hubei China

**Keywords:** Polycyclic aromatic hydrocarbons, Oxidative damage, Lung function, Mediation effect

## Abstract

**Background:**

Exposure to polycyclic aromatic hydrocarbons (PAHs) is related to decreased lung function. However, whether oxidative damage is involved in this relationship remains unclear. This study was aimed to explore the potential mediating role of oxidative DNA or lipid damage in the association between PAH exposure and lung function.

**Methods:**

The urinary levels of monohydroxy polycyclic aromatic hydrocarbon metabolites (OH-PAHs) and lung function parameters were measured among 3367 participants from the baseline of the Wuhan-Zhuhai cohort. Urinary 8-hydroxy-2′-deoxyguanosine (8-OHdG) and 8-isoprostane (8-*iso*-PGF2*α*) were determined to evaluate the individuals’ oxidative DNA and lipid damage degrees, respectively. Linear mixed models were used to investigate the associations of urinary OH-PAHs, 8-OHdG and 8-*iso*-PGF2*α* with lung function parameters. Mediation analysis was further conducted to assess the potential role of oxidative damage in the association between urinary OH-PAHs and lung function.

**Results:**

Each one-percentage increase in the sum of urinary OH-PAHs, high-molecular-weight or low-molecular-weight OH-PAHs (ƩOH-PAHs, ƩHMW OH-PAH or ƩLMW OH-PAHs, respectively) was associated with a 0.2152-, 0.2076- or 0.1985- ml decrease in FEV_1_, and a 0.1891-, 0.2195- or 0.1634- ml decrease in FVC, respectively. Additionally, significantly positive dose-response relationships of ƩOH-PAHs, ƩHMW OH-PAH and ƩLMW OH-PAHs with urinary 8-OHdG or 8-*iso*-PGF2*α*, as well as an inverse dose-response relationship between urinary 8-OHdG and FVC, were observed (all *P* for trend < 0.05). Mediation analysis indicated that urinary 8-OHdG mediated 14.22% of the association between ƩHMW OH-PAH and FVC.

**Conclusion:**

Higher levels of oxidative DNA damage might be involved in the decreased levels of FVC caused by high-molecular-weight PAH exposure.

## Background

Polycyclic aromatic hydrocarbons (PAHs) are a group of widespread environmental pollutants, primarily derived from incomplete combustion of fossil fuels and biomass [1]. Compared with natural exposure sources, industrial production processes, motor vehicle exhaust, cigarette smoking and residential fuel combustion are the main emission sources of PAHs in the human living environment [2]. In recent years, exposure to PAHs has been demonstrated to be associated with adverse health effects on various organs [3–7], especially the respiratory system [8, 9]. As an early indicator of respiratory damage, lung function can be used to predict the long-term morbidity and mortality of several diseases including nonrespiratory diseases [10, 11]. Additionally, a recent study of our research group has already discovered a significant association between urinary monohydroxy polycyclic aromatic hydrocarbons (OH-PAHs) and decreased lung function [8]. However, the underlying mechanisms remain incompletely understood.

Oxidative stress is an imbalance between oxidant and antioxidant capacity due to the overproduction of oxidative products [12] and is commonly considered to be involved in the pathogenesis of adverse health effects induced by PAH exposure [13, 14]. Accumulated oxidative products could initiate oxidative damage by attacking biological macromolecules in tissues, such as proteins, lipids and DNA [15]. Urinary 8-hydroxy-2′-deoxyguanosine (8-OHdG), one of the predominant forms of oxidative DNA lesions, is widely used as a biomarker for oxidative DNA damage in large-sample population studies [16, 17]. Similarly, urinary 8-isoprostane (8-*iso*-PGF2*α*), the terminal product of cell membrane lipidperoxidation with strong chemical stability, could reflect oxidative damage of lipids [18, 19]. Both epidemiology and toxicology studies have observed elevated levels of oxidative damage along with PAH exposure [12, 18, 20].

Although elevated levels of oxidative damage were reported in various airway diseases, such as asthma [21, 22], bronchiectasis [23], chronic obstructive pulmonary disease (COPD) [24–26] and idiopathic pulmonary fibrosis [27, 28], the relationship between oxidative damage and lung function alteration in healthy adults has been scarcely reported. Moreover, whether oxidative damage plays a potential role in lung function decline induced by PAH exposure remains largely unknown.

Thus, it is reasonable to assume that increased oxidative damage level may mediate the relationship between PAH exposure and lung function. In the present study, we determined urinary OH-PAHs as biomarkers for PAH exposure, measured urinary 8-OHdG and 8-*iso*-PGF2*α* levels as oxidative damage biomarkers and conducted lung function tests in 3367 participants from the baseline of the Wuhan-Zhuhai cohort. The associations of PAH exposure with lung function parameters and oxidative damage levels were assessed by using linear mixed models and restricted cubic spline regression models. Furthermore, mediation analysis was conducted to explore the role of oxidative damage biomarkers in the associations of lung function with PAH exposure.

## Methods

### Study population

This study was based on the Wuhan-Zhuhai (WHZH) cohort, which was described previously [29]. Briefly, 4812 adults dwelling in Wuhan or Zhuhai for more than 5 years were recruited. Before the physical examination, all the participants were informed to keep fasting for more than 12 h. During the investigation, they were required to complete the physical examinations and structured questionnaires and provide early-morning urine samples. The urine samples were collected in polypropylene containers and frozen until analysis. Detailed information on the demographic characteristics and lifestyle was obtained from questionnaires by face-to-face interviews. It included age, gender, education level, annual family income, ever occupational hazard exposure, smoking status, smoking amounts, passive smoking status, drinking status, cooking status, regular physical activity, sleep duration at night, diet information (including self-reported frequencies of food intake) and self-reported history of diseases.

In the current study, education levels were classified into three groups: low (middle school and below), middle (high school) and high (university or above). Annual family income was divided into three levels: < 30,000, 30,000–70,000 and ≥ 70,000 Yuan. Individuals who drank once a week for at least 6 months were defined as current drinkers and those who smoked at least one cigarette per day for more than 6 months were defined as current smokers. Smoking amounts (pack-years) for smokers were further calculated as packs of cigarettes per day multiplied by years of smoking. Passive smoking was defined as passive exposure to tobacco smoke for more than 1 day per week anywhere. Regular exercise within the last 6 months was considered as active physical activity. Self-reported respiratory diseases were defined as having at least one disease, including COPD, asthma, emphysema, chronic bronchitis, pneumoconiosis and pleurisy. Additionally, physical examinations were conducted by trained physicians. Body mass index (BMI) was calculated as weight (kg) divided by height (m) squared.

Excluding participants with self-reported respiratory diseases (*N* = 253) and potential occupational PAH exposure (*N* = 33), or with missing data on lung function index (*N* = 55), urinary OH-PAHs (*N* = 652), 8-OHdG (*N* = 344) and 8-*iso*-PGF2*α* (*N* = 56) as well as with missing data on life habits, such as sleep duration and diet information (*N* = 52), 3367 participants were included in the final analysis. The research protocol was approved by the Ethics and Human Subjects Committee of Tongji Medical College, Huazhong University of Science and Technology. Likewise, everyone signed written informed consent.

### Determination of urinary OH-PAHs

Twelve urinary OH-PAHs, including 1-hydroxypyrene (1-OHP), 6-hydroxychrysene (6-OHChr), 3-hydroxybenzo[a]pyrene (3-OHBaP), 1-hydroxynaphthalene (1-OHNa), 2-hydroxynaphthalene (2-OHNa), 2-hydroxyfluorene (2-OHFlu), 9-hydroxyfluorene (9-OHFlu), 1-hydroxyphenanthrene (1-OHPh), 2-hydroxyphenanthrene (2-OHPh), 3-hydroxyphenanthrene (3-OHPh), 4-hydroxyphenanthrene (4-OHPh) and 9-hydroxyphenanthrene (9-OHPh) were determined by gas chromatography-mass spectrometry (GC/MS; Agilent 6890 N + 59758B, Agilent Technologies Inc., Santa Clara, CA, USA) as previously reported by Li et al. [30]. Ten percent of urine samples were measured in duplicate for repeatability tests, and the coefficient of variation in the duplicate analysis was below 10%. Because the concentrations of 6-OHChr and 3-OHBaP were mostly below the limits of detection (LOD), the other 10 PAH metabolites remained in the final analysis. The LOD for urinary PAH metabolites ranged from 0.1 to 0.9 μg/l, and concentrations below the LOD were replaced by half the value of the LOD. Valid concentrations of OH-PAHs were calibrated by the levels of urinary creatinine (creat.) and expressed as μmol/mol creat, due to the urine dilution. For analysis, the sum of all OH-PAHs (ƩOH-PAHs) and sum of high- (≥4 rings, including 1-OHP) or low- (< 4 rings, including 1-OHNa, 2-OHNa, 2-OHFlu, 9-OHFlu, 1-OHPh, 2-OHPh, 3-OHPh, 4-OHPh and 9-OHPh) molecular-weight OH-PAHs (ƩHMW OH-PAH or ƩLMW OH-PAHs) were used in this study.

### Determination of urinary 8-OHdG

The urinary levels of 8-OHdG were measured using high-performance liquid chromatography (HPLC) coupled with an electrochemical detector (Waters 2645; Waters Inc., USA). The cleanup and analysis processes were performed as described previously [31]. The coefficient of variation was less than 5% in duplicate analysis, and the recoveries of spiked samples ranged from 75 to 120%. Similarly, the concentrations below the LOD were replaced by half of the LOD. Valid concentrations of 8-OHdG were calculated as μmol/mol creat.

### Determination of urinary 8-*iso*-PGF2*α*

The urinary concentrations of 8-*iso*-PGF2*α* were determined using a commercially available ELISA kit (Cayman, USA) following the manufacturer’s instructions (Catalog No. 516351). The LOD was approximately 2.7 pg/ml, and the levels of 8-*iso*-PGF2*α* were calibrated by urinary creat. and expressed as nmol/mol creat.

### Lung function test

Lung function parameters, including forced expiratory volume in 1 s (FEV_1_), forced vital capacity (FVC) and the ratio of FEV_1_ to FVC (FEV_1_%), were conducted by specialists using electronic spirometers (Chest graph HI-101; CHEST Ltd., Tokyo, Japan), according to the American Thoracic Society Recommendations, as described elsewhere [8]. All participants were informed to maintain normal breathing for at least 5 min in a sitting position with a nose clip in place before the test. Meanwhile, participants were advised not to smoke for at least 1 h and keep fasting for more than 2 h before the test. The greatest values for FEV_1_, FVC and FEV_1_% were obtained from multiple repeat measurements.

### Statistical analysis

The distributions of the basic characteristics were analyzed according to quartiles of urinary ƩOH-PAHs. Due to the right-skewed distributions and nonnormality of the residuals, the concentrations of urinary OH-PAHs, 8-OHdG and 8-*iso*-PGF2*α* were natural log-transformed before statistical analysis. Both continuous and categorical variable models were conducted to quantify the associations of urinary OH-PAHs with oxidative damage biomarkers (8-OHdG and 8-*iso*-PGF2*α*) and lung function parameters by linear mixed models and restricted cubic spline regression models. All models were adjusted for age (continuous variable), gender (male/female), height (continuous variable), weight (continuous variable), smoking amounts (continuous variable), passive smoking status (yes/no), drinking status (yes/no), education level (categorical variable), annual family income (categorical variable), regular physical activity (yes/no), cooking meals at home (yes/no), sleep duration at night (continuous variable), eating smoked food (< 1/≥1 time/week), eating vegetables or fruits (< 1/≥1 time/day), eating aquatic products (< 1/≥1 time/day) and city (Wuhan/Zhuhai). Stratified analyses were also conducted both in nonsmokers and smokers to ascertain the above associations.

Additionally, mediation analysis was performed to assess the mediating role of oxidative damage biomarkers in the associations between urinary OH-PAHs and lung function. We used linear mixed models to explore the associations of exposure-outcome (OH-PAHs and lung function), exposure-mediator (OH-PAHs and oxidative damage biomarkers) and exposure-mediator-outcome (OH-PAHs, oxidative damage biomarkers and lung function) (Eqs (1) to (3)), respectively.

1 $$ \mathrm{Y}={\alpha}_0+{\varphi}_0+{\alpha}_1{X}_1+{\alpha}_2{X}_2+\cdots +{\alpha}_{OH- PAHs}{X}_{OH- PAHs}+\omega $$

2 $$ \mathrm{M}={\upbeta}_0+{\varepsilon}_0+{\beta}_1{X}_1+{\beta}_2{X}_2+\cdots +{\beta}_{OH- PAHs}{X}_{OH- PAHs}+\mu $$

3 $$ {Y}^{\prime }={\gamma}_0+{\delta}_0+{\gamma}_1{X}_1+{\gamma}_2{X}_2+\cdots +{\gamma}_{OH- PAHs}{X}_{OH-\mathrm{P} AHs}+{\gamma}_MM+\rho $$

In each equation, X = independent variable (including OH-PAHs and covariates), M = mediator (oxidative damage biomarkers), and Y (or Y′) = dependent variable (lung function parameters). *α*_*OH* − *PAHs*_ presents the total effect, *γ*_*OH* − *PAHs*_ presents the direct effect, and the mediated effect is calculated as the product of *β*_*OH* − *PAHs*_ and *γ*_*M*_.

We also performed a mediation test to calculate confidence limits for the mediated effects of oxidative damage using the PROCESS SPSS macro [32]. Additionally, the proportion mediated is calculated as the ratio of the mediation effect to the total effect. All analyses were performed using SAS version 9.4 (SAS institute, Cary, NC, USA) and SPSS version 17.0 (SPSS Inc., Chicago, IL, USA).

## Results

The basic characteristics of the participants by quartiles of urinary ƩOH-PAHs are shown in Table 1. In the present study, the mean age of all 3367 participants was 52.0 years, and one-third were males (31.0%). The participants with higher levels of urinary ƩOH-PAHs were more likely to be females, older and to engage in regular physical activity, cook meals or eat smoked food and were less likely to be smokers, drinkers and to have a higher BMI; however, the smoking amounts of smokers were significantly elevated with higher quartiles of urinary ƩOH-PAHs. Moreover, as the concentrations of urinary ƩOH-PAHs increased, the average values of FEV_1_ and FVC were monotonically decreased; whereas the levels of 8-OHdG and 8-*iso*-PGF2*α* were significantly increased.

**Table 1:** Characteristics of participants by quartiles of urinary OH-PAH levels (*N* = 3367)

Characteristics	All participants	Quartiles of ƩOH-PAHs, μmol/mol creat.			
		Quartile 1 (< 24.63)	Quartile 2 (24.63–36.12)	Quartile 3 (36.12–54.86)	Quartile 4 (≥54.86)
No. participants	3367	842	842	842	841
Age (years, means±SD)	52.0 ± 12.9	49.9 ± 13.2	51.1 ± 12.8	53.0 ± 12.4	53.9 ± 12.8
Gender (male, N, %)	1042 (31.0)	336 (39.9)	285 (33.9)	241 (28.6)	180 (21.4)
Height (cm, means±SD)	159.0 ± 7.7	160.6 ± 8.0	159.3 ± 7.3	158.7 ± 7.5	157.6 ± 7.7
Weight (kg, means±SD)	60.8 ± 10.5	62.5 ± 11.0	61.0 ± 10.8	60.7 ± 9.9	58.8 ± 9.8
BMI (kg/m^2^, means±SD)	24.0 ± 3.4	24.2 ± 3.5	24.0 ± 3.5	24.1 ± 3.3	23.7 ± 3.5
Education level (N, %)					
Low	828 (24.6)	190 (22.6)	204 (24.2)	236 (28.0)	198 (23.5)
Middle	2122 (63.0)	540 (64.1)	539 (64.0)	514 (61.1)	529 (62.9)
High	417 (12.4)	112 (13.3)	99 (11.8)	92 (10.9)	114 (13.6)
Annual family income (Yuan, N, %)					
< 30,000	1924 (57.1)	458 (54.4)	492 (58.4)	497 (59.0)	477 (56.7)
30,000-70,000	1091 (32.4)	270 (32.1)	277 (32.9)	272 (32.3)	272 (32.3)
≥70,000	352 (10.5)	114 (13.5)	73 (8.7)	73 (8.7)	92 (10.9)
Smokers^a^ (N, %)	740 (21.6)	196 (23.3)	193 (22.9)	189 (22.5)	147 (17.5)
Smoking amounts^b^, pack-year (means±SD)	24.7 ± 21.3	20.1 ± 21.1	24.7 ± 19.5	27.9 ± 22.2	26.9 ± 21.8
Passive smokers (yes, N, %)	1451 (43.1)	346 (42.4)	357 (42.4)	399 (47.5)	349 (41.5)
Cook meals (yes, N, %)	2500 (74.3)	577 (68.5)	617 (73.3)	644 (76.5)	662 (78.7)
Drinking status (yes, N, %)	577 (17.1)	149 (17.7)	169 (20.1)	147 (17.5)	112 (13.3)
Regular physical activity (yes, N, %)	1579 (46.9)	393 (46.7)	373 (44.3)	386 (45.8)	427 (50.8)
Sleep duration at night (hours, means±SD)	8.0 ± 1.4	8.0 ± 1.7	8.0 ± 1.3	8.1 ± 1.4	7.9 ± 1.3
Eating smoked food (≥1 time/week, N, %)	1009 (30.0)	213 (25.3)	250 (29.7)	280 (33.3)	266 (31.6)
Eating vegetables or fruits (≥1 time/day, N, %)	3163 (93.9)	802 (95.3)	791 (93.9)	785 (93.2)	785 (93.3)
Eating aquatic products (≥1 time/day, N, %)	1128 (33.5)	294 (34.9)	294 (34.9)	277 (32.9)	263 (31.3)
FEV_1_ (ml, means±SD)	2189.4 ± 586.1	2327.7 ± 625.5	2215.6 ± 582.0	2160.3 ± 547.8	2053.9 ± 553.1
FVC (ml, means±SD)	2508.8 ± 682.5	2640.3 ± 730.7	2535.7 ± 675.2	2497.0 ± 645.0	2358.6 ± 646.8
FEV_1_% (means±SD)	87.7 ± 8.4	88.7 ± 8.0	87.8 ± 8.0	87.0 ± 8.6	87.5 ± 8.9
8-OHdG (μmol/mol creat., median, IQR)	62.2 (28.0–123.7)	48.0 (21.9–90.6)	60.6 (26.9–114.0)	66.0 (727.9–129.8)	84.4 (41.1–179.3)
8-*iso*-PGF2*α* (nmol/mol creat., median, IQR)	177.0 (108.9–311.7)	126.3 (81.3–195.0)	167.0 (104.1–276.4)	207.9 (129.9–348.1)	240.8 (139.4–504.6)

*Abbreviations*: *ƩOH-PAHs* Sum of urinary monohydroxy polycyclic aromatic hydrocarbons, 8-*iso*-PGF2*α* 8-isoprostane, *8-OHdG* 8-hydroxy-2′-deoxyguanosine, *BMI* Body mass index, *FEV*_*1*_ Forced expiratory volume in 1 s, *FVC* Forced vital capacity, *FEV*_*1*_*%* The ratio of FEV_1_ to FVC, *IQR* Interquartile range, *SD* Standard deviation

^a^Smokers included current and former smokers

^b^Smoking amounts were calculated as packs of cigarettes per day multiplied by years of smoking

As displayed in Table 2, each one-percentage increase in urinary ƩOH-PAHs, ƩHMW OH-PAH or ƩLMW OH-PAHs generated a 0.2152-, 0.2076- or 0.1985- ml decrease in FEV_1_, respectively (all *P* < 0.01). Only each one-percentage increase in ƩHMW OH-PAH was found to be significantly associated with a − 0.2159 ml change in FVC. We also found a marginal association of FVC with ƩOH-PAHs or ƩLMW OH-PAHs. In the categorical variable models, FEV_1_ was monotonically decreased with elevated quartiles of urinary ƩOH-PAHs, ƩHMW OH-PAH or ƩLMW OH-PAHs (all *P* for trend < 0.05). A similar trend was observed for FVC with ƩHMW OH-PAH. Additionally, no significant relationship was observed between urinary OH-PAHs (including ƩOH-PAHs, ƩHMW OH-PAH and ƩLMW OH-PAHs) and FEV_1_% (all *P* > 0.05).

**Table 2:** Associations between urinary OH-PAHs and lung function parameters (*N* = 3367)

Urinary OH-PAHs	Lung function parameters	Estimated changes (95% CI) by continuous OH-PAHs	Estimated changes in ml (95% CI) by quartile of OH-PAHs				*P* value for trend^a^
			Quartile 1	Quartile 2	Quartile 3	Quartile 4	
ƩOH-PAHs			< 24.63	24.63–36.12	36.12–54.86	≥54.86	
	FEV_1_	−21.52 (−40.46, −2.58)	0 (referent)	−39.63 (−76.83, −2.43)	−11.74 (− 49.41, 25.94)	−65.59 (− 103.42, − 27.77)	0.0057
	FVC	− 18.91 (− 41.95, 4.14)	0 (referent)	− 34.69 (−79.99, 10.61)	2.51 (−43.39, 48.42)	−57.89 (− 103.95, − 11.83)	0.0651
	FEV_1_%	−0.17 (−0.57, 0.23)	0 (referent)	−0.49 (− 1.28, 0.29)	− 0.53 (− 1.32, 0.27)	− 0.57 (− 1.37, 0.23)	0.1791
ƩHMW OH-PAH			< 1.99	1.99–3.21	3.21–5.41	≥5.41	
	FEV_1_	−20.76 (− 37.69, − 3.83)	0 (referent)	−25.06 (−61.93, 11.81)	− 31.01 (− 68.12 6.11)	−54.55 (−91.82, − 17.28)	0.0049
	FVC	− 21.59 (−42.18, − 1.00)	0 (referent)	− 9.94 (− 54.77, 34.89)	−31.06 (− 76.20, 14.08)	−54.59 (− 99.91, − 9.27)	0.0116
	FEV_1_%	− 0.07 (− 0.43, 0.29)	0 (referent)	− 0.58 (− 1.36, 0.19)	−0.36 (− 1.15, 0.42)	−0.30 (− 1.08, 0.49)	0.5963
ƩLMW OH-PAHs			< 21.68	21.68–32.24	32.24–49.23	≥49.23	
	FEV_1_	−19.85 (− 38.44, − 1.26)	0 (referent)	−39.60 (− 76.90, − 12.31)	−15.47 (−53.18, 22.24)	− 61.75 (−99.81, − 23.70)	0.0090
	FVC	−16.34 (− 38.60, 6.27)	0 (referent)	−37.59 (− 83.01, 7.82)	−3.39 (− 49.34, 42.56)	−50.65 (− 97.01, −4.30)	0.1166
	FEV_1_%	− 0.19 (− 0.59, 0.20)	0 (referent)	− 0.36 (− 1.15, 0.43)	−0.42 (− 1.22, 0.38)	−0.65 (− 1.46, 0.15)	0.1205

Model adjusted for age (continuous variable), gender (male/female), height (continuous variable), weight (continuous variable), smoking amounts (continuous variable), passive smoking status (yes/no), drinking status (yes/no), education level (categorical variable), annual family income (categorical variable), regular physical activity (yes/no), cooking meals at home (yes/no), sleep duration at night (continuous variable), eating smoked food (< 1/≥1 time/week), eating vegetables or fruits (< 1/≥1 time/day), eating aquatic products (< 1/≥1 time/day) and city (Wuhan/Zhuhai)

*Abbreviations*: *ƩHMW OH-PAH* Sum of urinary high-molecular-weight monohydroxy polycyclic aromatic hydrocarbon, including 1-hydroxypyrene, *ƩLMW OH-PAHs* Sum of urinary low-molecular-weight monohydroxy polycyclic aromatic hydrocarbon, including 1-hydroxynaphthalene, 2-hydroxynaphthalene, 2-hydroxyfluorene, 9-hydroxyfluorene, 1-hydroxyphenanthrene, 2-hydroxyphenanthrene, 3-hydroxyphenanthrene, 4-hydroxyphenanthrene and 9-hydroxyphenanthrene, *ƩOH-PAHs* Sum of urinary monohydroxy polycyclic aromatic hydrocarbons, *CI* Confidence interval, *FEV*_*1*_ Forced expiratory volume in 1 s, *FVC* Forced vital capacity, *FEV*_*1*_% The ratio of FEV_1_ to FVC

^a^*P* trend values of the quartile coefficients were estimated by including the original log-transformed OH-PAHs as a continuous variable

**Fig. 1:**
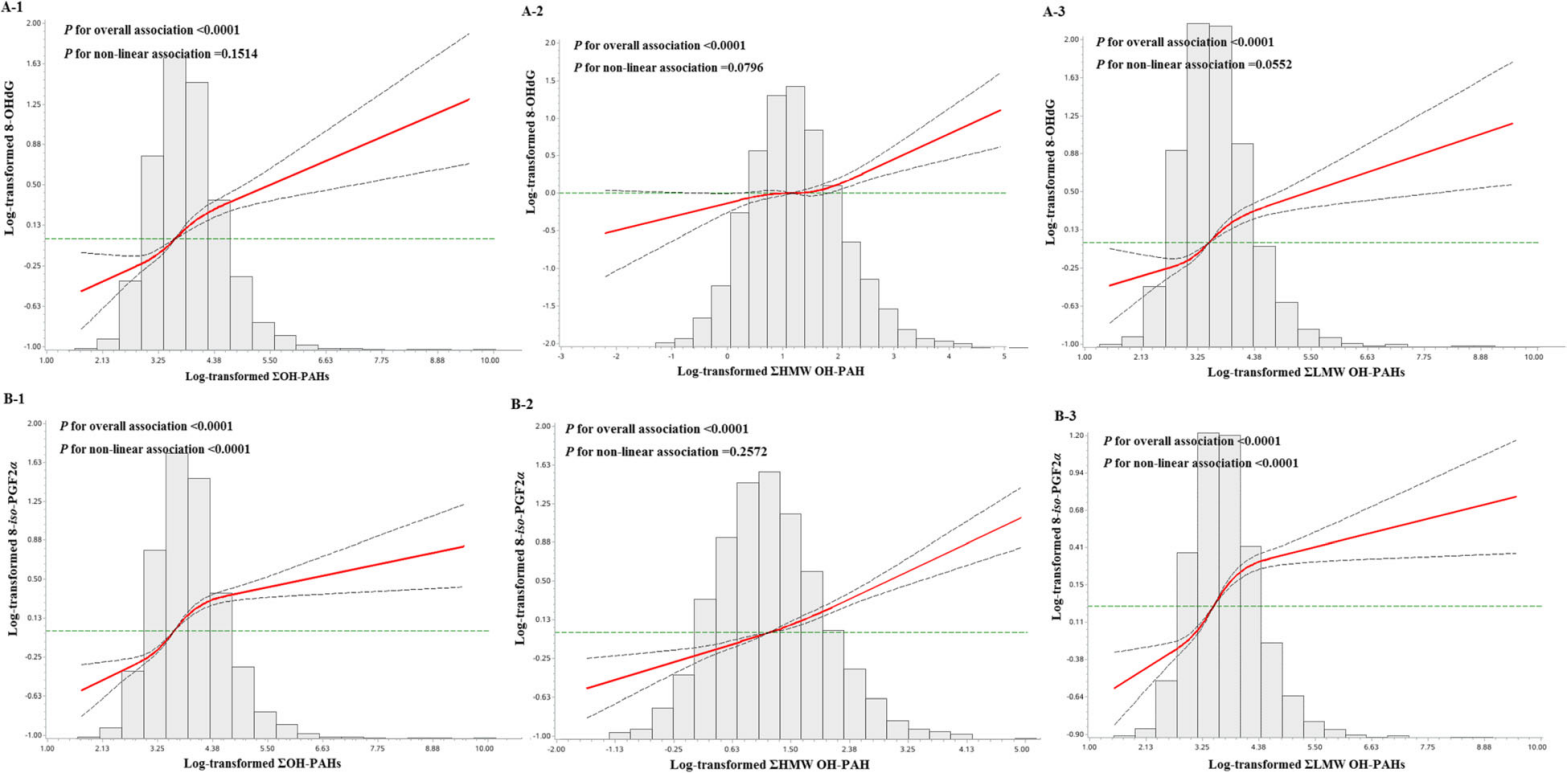
Restricted cubic splines representing the associations of urinary OH-PAHs with 8-OHdG (**a**) and 8-*iso*-PGF2*α* (**b**), with adjustment for age (continuous variable), gender (male/female), height (continuous variable), weight (continuous variable), smoking amounts (continuous variable), passive smoking status (yes/no), drinking status (yes/no), education level (categorical variable), annual family income (categorical variable), regular physical activity (yes/no), cooking meals at home (yes/no), sleep duration at night (continuous variable), eating smoked food (< 1/≥1 time/week), eating vegetables or fruits (< 1/≥1 time/day), eating aquatic products (< 1/≥1 time/day) and city (Wuhan/Zhuhai). Knots were placed at the 5th, 35th, 65th 95th percentiles of the independent variables distributions, and the reference value was set at the 5th percentile. Abbreviations: ƩHMW OH-PAH, sum of urinary high-molecular-weight monohydroxy polycyclic aromatic hydrocarbon, including 1-hydroxypyrene; ƩLMW OH-PAHs, sum of urinary low-molecular-weight monohydroxy polycyclic aromatic hydrocarbon, including 1-hydroxynaphthalene, 2-hydroxynaphthalene, 2-hydroxyfluorene, 9-hydroxyfluorene, 1-hydroxyphenanthrene, 2-hydroxyphenanthrene, 3-hydroxyphenanthrene, 4-hydroxyphenanthrene and 9-hydroxyphenanthrene; ƩOH-PAHs, sum of urinary monohydroxy polycyclic aromatic hydrocarbons; 8-*iso*-PGF2*α,* 8-isoprostane; 8-OHdG, 8-hydroxy-2′-deoxyguanosine; OH-PAHs, urinary monohydroxy polycyclic aromatic hydrocarbons

Figure 1 presents the associations of urinary OH-PAHs with 8-OHdG and 8-*iso*-PGF2*α*. We observed that significant monotonically elevated levels of 8-OHdG or 8-*iso*-PGF2*α*, as the levels of urinary ƩOH-PAHs, ƩHMW OH-PAH and ƩLMW OH-PAHs increased. Additionally, a nonlinear relationship was revealed between urinary 8-*iso*-PGF2*α* and urinary ƩOH-PAHs or ƩLMW OH-PAHs (*P* < 0.0001). Considering that cigarette smoke could induce oxidative damage in humans, stratified analyses by smoking status were further conducted to avoid its interference. The associations between urinary OH-PAHs and 8-OHdG or 8-*iso*-PGF2*α *in both nonsmokers and smokers were similar to those of the whole population (data not shown).

The associations between oxidative damage biomarkers and lung function are revealed in Table 3. In continuous variable analysis, each one-percentage increase in urinary 8-OHdG was significantly associated with a 0.1748 ml decrease in FVC. The categorical variable model only showed a significantly negative dose-response relationship of urinary 8-OHdG with FVC (*P* for trend =0.0430). No significant association was found between urinary 8-*iso*-PGF2*α* and any of these lung function parameters.

**Table 3:** Associations between oxidative damage and lung function parameters (*N* = 3367)

Oxidative damage	Lung function parameters	Estimated changes (95% CI) by continuous OH-PAHs	Estimated changes in ml (95% CI) by quartile of oxidative damage				*P* value for trend^a^
			Quartile 1	Quartile 2	Quartile 3	Quartile 4	
8-OHdG			< 28.79	28.79–63.18	63.18–125.04	≥125.04	
	FEV_1_	−6.08 (− 17.28, 5.11)	0 (referent)	−8.51 (− 45.57, 28.55)	− 10.00 (− 47.14, 27.15)	−21.92 (− 59.30, 15.45)	0.2651
	FVC	− 17.48 (− 31.08, − 3.88)	0 (referent)	− 16.98 (− 62.01, 28.06)	−17.65 (− 62.79, 27.49)	−46.03 (− 90.65, − 1.41)	0.0430
8-*iso*-PGF2*α*			< 109.09	109.09–176.89	176.89–310.79	≥310.79	
	FEV_1_	−11.31 (− 29.56, 6.93)	0 (referent)	3.25 (−32.62, 39.13)	−8.71 (− 44.37, 26.96)	−13.57 (− 50.12, 22.98)	0.4241
	FVC	−16.15 (− 38.38, 6.07)	0 (referent)	3.57 (−40.06, 47.20)	− 19.90 (− 63.26, 23.45)	−26.93 (− 71.51, 17.65)	0.1813

Model adjusted for age (continuous variable), gender (male/female), height (continuous variable), weight (continuous variable), smoking amounts (continuous variable), passive smoking status (yes/no), drinking status (yes/no), education level (categorical variable), annual family income (categorical variable), regular physical activity (yes/no), cooking meals at home (yes/no), sleep duration at night (continuous variable), eating smoked food (< 1/≥1 time/week), eating vegetables or fruits (< 1/≥1 time/day), eating aquatic products (< 1/≥1 time/day) and city (Wuhan/Zhuhai)

*Abbreviations*: 8-*iso*-PGF2*α* 8-isoprostane, *8-OHdG* 8-hydroxy-2′-deoxyguanosine, *CI* Confidence interval, *FEV*_*1*_ Forced expiratory volume in 1 s, *FVC* Forced vital capacity

^a^*P* trend values of the quartile coefficients were estimated by including the original log-transformed oxidative damage as a continuous variable

Table 4 shows the associations between urinary OH-PAHs regarding lung function parameters and the mediation assessment of urinary 8-OHdG among the relationships. It showed that significant mediated effect of 8-OHdG in the models associating ƩHMW OH-PAH with FVC. The direct effect of urinary ƩHMW OH-PAH on FVC was − 18.30 ml (− 39.07 ml, 2.47 ml). Additionally, the mediated proportion by 8-OHdG in the relationship between ƩHMW OH-PAH and FVC was 14.22%. However, no significant mediation effect of 8-OHdG was observed regarding the relationships between PAH exposures and FEV_1_, as well as between ƩLMW OH-PAHs and FVC.

**Table 4:** Total and direct effect of urinary OH-PAHs on lung function alteration and assessment of 8-OHdG (*N* = 3367)

	Total effects^a^	Direct effects^b^	Mediated effect of 8-OHdG^c^	Proportion mediated (%)
FEV_1_				
ƩOH-PAHs	−21.76 (− 40.74, − 2.79)	−20.52 (− 39.80, − 1.25)	−1.24 (− 4.78, 2.74)	NA^d^
ƩHMW OH-PAH	− 20.49 (− 37.44, − 3.53)	− 19.61 (− 36.69, − 2.52)	− 0.88 (− 3.22, 1.48)	NA^d^
ƩLMW OH-PAHs	−20.19 (−38.81, − 1.57)	−18.93 (− 37.85, − 0.02)	−1.26 (− 5.07, 2.15)	NA^d^
FVC				
ƩOH-PAHs	− 18.93 (− 42.02, 4.16)	− 14.10 (− 37.54, 9.34)	−4.83 (− 9.89, − 0.45)	NA^d^
ƩHMW OH-PAH	−21.33 (− 41.96, − 0.71)	−18.30 (− 39.07, 2.47)	−3.03 (− 6.23, − 0.57)	14.22%
ƩLMW OH-PAHs	− 16.46 (− 39.12, 6.20)	− 11.66 (− 34.66, 11.35)	−4.80 (− 9.62, − 0.66)	NA^d^

Covariates in the SPSS commands include age (continuous variable), gender (male/female), height (continuous variable), weight (continuous variable), smoking amounts (continuous variable), passive smoking status (yes/no), drinking status (yes/no), education level (categorical variable), annual family income (categorical variable), regular physical activity (yes/no), cooking meals at home (yes/no), sleep duration at night (continuous variable), eating smoked food (< 1/≥1 time/week), eating vegetables or fruits (< 1/≥1 time/day), eating aquatic products (< 1/≥1 time/day) and city (Wuhan/Zhuhai)

*Abbreviations*: *ƩHMW OH-PAH* Sum of urinary high-molecular-weight monohydroxy polycyclic aromatic hydrocarbon, including 1-hydroxypyrene, *ƩLMW OH-PAHs* Sum of urinary low-molecular-weight monohydroxy polycyclic aromatic hydrocarbon, including 1-hydroxynaphthalene, 2-hydroxynaphthalene, 2-hydroxyfluorene, 9-hydroxyfluorene, 1-hydroxyphenanthrene, 2-hydroxyphenanthrene, 3-hydroxyphenanthrene, 4-hydroxyphenanthrene and 9-hydroxyphenanthrene, *ƩOH-PAHs* Sum of urinary monohydroxy polycyclic aromatic hydrocarbons, *8-OHdG* 8-hydroxy-2′-deoxyguanosine, *CI* Confidence interval, *FEV*_*1*_ Forced expiratory volume in 1 s, *FVC* Forced vital capacity

^a^Total effects of urinary OH-PAHs on lung function parameters were estimated without adjusting for urinary 8-OHdG

^b^Direct effects of urinary OH-PAHs on lung function parameters were estimated with adjusting for urinary 8-OHdG

^c^The mediated effect of 8-OHdG was obtained from the model, which simultaneously included OH-PAHs, 8-OHdG, lung function parameters and covariates by performing in the PROCESS SPSS macro

^d^Proportion mediated by 8-OHdG cannot be tested because of the opposite signs between total effect and indirect effect

## Discussion

In this study, we found that urinary OH-PAHs were significantly negatively associated with lung function, and positively related to oxidative damage in DNA or lipids. Additionally, inverse dose-response relationships were further observed between urinary 8-OHdG and FVC, indicating that higher 8-OHdG levels might be a risk factor for decreased lung function. Moreover, our results suggest that urinary 8-OHdG may partially mediate the association between urinary ΣHMW OH-PAH and FVC.

Along with our previous study [8], the associations between PAH exposure and altered lung function have been widely conducted in occupational [33], pediatric [34, 35] and general populations [36]. It is generally believed that oxidative stress plays a pivotal role in the pathogenic process following PAH exposure. Consistent with our findings, epidemiological studies have observed significant dose-response relationships between urinary OH-PAHs and oxidative damage in occupational [18, 37] and general populations [12]. To avoid possible interference from smoking, which is a strong oxidant [38, 39], we further evaluated such relationships in both smokers and nonsmokers and obtained similar results. Our findings suggest that PAH exposure is significantly associated with oxidative damage, regardless of cigarette smoking.

Meanwhile, the effects of PAH exposure on oxidative stress were also reported in studies performed in vivo and in vitro [13, 14]. After being absorbed in the body, PAHs from various sources can be metabolized into active semiquinones via cytochrome P450 enzymes; the free radical intermediates could further generate reactive oxygen species (ROS). Exaggerated activation of ROS could cause oxidative modification of DNA and lipids in lung tissue [15, 40], further contributing to lung function reduction.

Furthermore, higher levels of oxidative damage were reported in various respiratory diseases, but few studies have investigated the associations between oxidative damage and lung function. Several studies have reported that oxidative stress is inversely correlated with lung function parameters in COPD patients compared with healthy controls in the USA [41], Japan [42] and China [43]. However, a cross-sectional study from Australia showed that urinary 8-OHdG of quartz-exposed workers was positively correlated with FEV_1_ and FVC in the case of silicosis [44]. The discrepancy may be partly explained by the different types of lung diseases and the process of DNA damage and repair status. Regarding general populations, only one adolescent study from Italy showed that urinary 8-*iso*-PGF2*α* was negatively correlated with the respiratory flux index, including FEF_50_, FEF_25–75_ and FEV_1_% [45]. Partly consistent with previous studies, we found that an increased level of 8-OHdG was associated with decreased lung function in a general population after excluding those with diagnosed lung disease; however, we found no significant relationships between urinary 8-*iso*-PGF2*α* and lung function parameters.

As an important lung function parameter, FVC can help determine both the presence and severity of restrictive airway diseases. Airway epithelial cells directly play as a natural barrier to prevent inhaled xenobiotics or particulate matter [46]. Sustained exposure to ROS could induce DNA damage in epithelial cells, triggering apoptotic pathways through the upregulation of *p*53 and transforming growth factor *β* (TGF-*β*), and secretion of multiple factors from fibroblasts [47]. Increased apoptosis of pulmonary epithelial cells may cause the loss of balance in cell turnover, and the factors induced by fibroblasts contribute to the abnormal reepithelialization. Both processes are involved in the generation of fibrosis, leading to lung compliance decline and restrictive lung function impairment [48]. Moreover, enhanced oxidative stress has been found in lung epithelial cells of patients with idiopathic interstitial pneumonia, a condition that induce DNA damage and apoptosis [49].

Although both ƩHMW OH-PAH and ƩLMW OH-PAHs were found to be associated with oxidative damage and lung function parameters, our results indicated that the pathological effects of different molecular weight PAH exposures on lung injury might be different. It is well-known that low-molecular-weight PAHs are low in toxicity and noncarcinogenic; while high-molecular-weight PAHs, such as pyrene, the parent PAH of 1-OHP, demonstrate high toxic, carcinogenic and mutagenic activities [2]. In the present study, we found that urinary 8-OHdG significantly mediated the associations between high-molecular-weight PAH metabolites and FVC. Such a mediating role of 8-OHdG was not found in the association between low-molecular-weight PAH metabolites and lung function parameters. Relatively high oxidative DNA damage induced by high-molecular-weight PAH may contribute to this effect.

Our study has several strengths. First, the present study was conducted using a relatively large study population of 3367 participants. Second, we measured 10 types of urinary OH-PAHs, which could reflect internal PAH exposure levels from all potential sources. Additionally, choosing urinary 8-OHdG and 8-*iso*-PGF2*α* to represent systemic oxidative damage levels is a more comprehensive approach that, may provide a clue to help interpret the potential mechanism. One limitation in our study is that determining the levels of OH-PAH in a single spot urine sample might not reflect the long-term exposure levels of PAHs, although the life habits (including dietary pattern, travel mode, smoking status and other potential exposure routes for PAHs) of the study population were relatively stable. Further prospective studies are warranted. Furthermore, except for PAHs, exposure to other unmeasured toxic substances in the environment may also cause lung damage. Thus, the synergistic effects of multiple pollutants should be considered in further research. In addition, because 8-OHdG is a nonspecific biomarker for oxidative stress in lung tissue, to clarify the etiological pathway, it is necessary to identify more specific biomarkers.

## Conclusion

Urinary OH-PAHs levels were negatively associated with lung function, but positively related to the levels of oxidative stress. Moreover, our findings suggest that urinary 8-OHdG may play an important role in the association between high-molecular-weight PAH exposure and FVC, further providing a clue to the potential underlying mechanism of lung damage related to PAH exposure. More studies are needed to identify specific biomarkers to elucidate this damage process.

## Data Availability

The datasets generated and analyzed during the present study are not publicly available due to privacy concerns, as they contain sensitive and protected health data on participants. Specific requests to access the data can be sent to wchen@mails.tjmu.edu.cn.

## Abbreviations

ƩHMW OH-PAH: Sum of high-molecular-weight monohydroxy polycyclic aromatic hydrocarbon
ƩLMW OH-PAHs: Sum of low-molecular-weight monohydroxy polycyclic aromatic hydrocarbons
ƩOH-PAHs: Sum of urinary monohydroxy polycyclic aromatic hydrocarbons
1-OHNa: 1-hydroxynaphthalene
1-OHP: 1-hydroxypyrene
1-OHPh: 1-hydroxyphenanthrene
2-OHFlu: 2-hydroxyfluorene
2-OHNa: 2-hydronaphthalene
2-OHPh: 2-hydroxyphenanthrene
3-OHPh: 3-hydroxyphenanthrene
4-OHPh: 4-hydroxyphenanthrene
8-*iso*-PGF2α: 8-isoprostane
8-OHdG: 8-hydroxy-2′-deoxyguanosine
9-OHFlu: 9-hydroxyfluorene
9-OHPh: 9-hydroxyphenanthrene
BMI: Body mass index
CI: Confidence interval
creat.: Creatinine
FEV_1_: Forced expiratory volume in 1 s
FVC: Forced vital capacity
FEV_1_%: The ratio of FEV_1_ to FVC
GC-MS: Gas chromatography-mass spectrometry
HPLC: High-performance liquid chromatography
IQR: Interquartile range
LOD: The limits of detection
OH-PAHs: Monohydroxy polycyclic aromatic hydrocarbons metabolites
PAHs: Polycyclic aromatic hydrocarbons
SD: Standard deviation
